# Excess Mortality Attributable to *Clostridium difficile* and Risk Factors for Infection in an Historic Cohort of Hospitalised Patients Followed Up in the United Kingdom Death Register

**DOI:** 10.1371/journal.pone.0149983

**Published:** 2016-03-21

**Authors:** Mark Reacher, Neville Q. Verlander, Iain Roddick, Cheryl Trundle, Nicholas Brown, Mark Farrington, Philip Jones

**Affiliations:** 1 Cambridge Institute of Public Health, Public Health England, National Infection Service, Field Epidemiology Service, Cambridge, United Kingdom; 2 Clinical Microbiology and Public Health Laboratory (Public Health England), Addenbrooke's Hospital, Cambridge, United Kingdom; 3 Statistics unit, Statistics, Modelling and Economics Department, Public Health England, National Infection Service, Colindale, London, United Kingdom; 4 Department of Medical Microbiology, Ipswich Hospital, Ipswich, United Kingdom; Cleveland Clinic, UNITED STATES

## Abstract

**Methods:**

We compared time from hospital admission to death in a probability sample of 100 *Clostridium difficile* infected cases and a probability sample of 98 non-cases admitted to an English teaching hospital between 2005 and 2007 with follow up in the UK national death register using survival analysis.

**Results:**

*Clostridium difficile* infection was associated with a 50% increased risk of death (Hazard Ratio 1.51 (95% CI: 1.05–2.19 p = 0.03) at between five to eight years in Cox Regression analysis adjusting for age, sex, Charlson comorbidity index, diagnosis of a malignant condition and insertion of a nasogastric tube during admission. Acquisition of *Clostridium difficile* infection was independently associated with an almost six fold higher odds of being admitted with a diagnosis of infection of any other type (OR 5.79 (2.19, 15.25) p<0.001).

**Conclusions:**

Our results strongly support continued priority being given to improve prevention and treatment of *Clostridium difficile* infection in the English National Health Service particularly in patients admitted with an infection. Our results may be applicable to other health systems.

## Introduction

*Clostridium difficile* remains a leading cause of health care associated infectious diarrhoea worldwide. Transmission is by the faecal-oral route leading to ingestion of spores of toxin producing strains, which proliferate in the gut, to give disease, generally following antibiotic treatment, which is believed to supress the normal gut flora [1;2].

Mandatory reports of *Clostridium difficile* from hospitals in England peaked at 55,498 (108 per 100,000) in financial year 2007/2008 declining to 13, 361 (25 per 100,000) in 2013/2014, but with recent plateauing of the downward trend [3].

*Clostridium difficile* associated disease (CDAD) ranges from mild self-limiting, to severe protracted diarrhoea, dehydration, shock, sepsis, pseudo membranous colitis, toxic mega-colon and acute death [1].

Although the role of severe CDAD in acute death is well defined, [2] it has been difficult to determine the true attributable mortality of *Clostridium difficile* infection [4]. Incompleteness of studies of CDAD and mortality include relatively short term follow up; hospital based follow up (which may be less complete than follow up in a national death register); uncertain or absent reference groups to measure expected mortality in non-cases drawn from the same population at risk as cases; uncertain or absent adjustment for comorbidity, social deprivation and tobacco and alcohol use, which are themselves powerfully related to life expectancy [2;4–9]. Additionally, publication bias may favour reports of large and severe outbreaks, which may be associated with exceptionally virulent strains of *Clostridium difficile* with higher mortality than the general case mix of *Clostridium difficile* infections.

In order to measure if *Clostridium difficile* infection is associated with change in life expectancy in cases representative of all incident cases, we undertook an historic cohort study of a probability sample of cases and a probability sample of non-cases admitted between 1 Jan 2005 and 31 December 2007 to the same medical specialities, and therefore to the same wards, in Addenbrooke’s Hospital. We abstracted clinical information from structured review of the clinical notes and admission details from the Patient Administration System. We linked these to individuals’ death certificates identified by computer search of the UK national death register. To ensure all potentially relevant factors were included in the survival analysis and to give insight into the representativeness of our study population, we also undertook an analysis of risk factors for *Clostridium difficile* infection.

### Ethical and Institutional approvals

#### Cambridgeshire 3 Research Ethics Committee. Reference 09/H0306/62 Approved 17 August 2009

Application was made using the UK Integrated Research System (IRAS) and presented to the Committee by MR and PJ on 6 August 2009.

#### National Information Governance Board (NIGB ECC 6-06(g)/2009). Approved 22 September 2010

Because the study protocol required linkage of individual patient clinical records with death certificates in the UK National Death Register without consent from patients or their relatives, an additional ethical application was required to the National Information Governance Board under section 251- Control of patient information—of the UK National Health Service Act 2006. This application was made to the National Information Governance Board on 8 September 2009.

Conditional approval was given by the National Information Governance Board by letter dated 8 December 2009 subject to undertaking a survey of Addenbrooke’s Hospital users on the acceptability of the protocol from a patient perspective; and submission of a Systems Level Security Policy compliant with National Information Governance Board standards.

#### Survey of opinion of Addenbrooke’s Hospital patient users

A survey of the acceptability of the study design to Addenbrooke’s patient users was conducted by the investigators with the assistance of the Cambridge University Hospitals NHS Foundation Trust Research and Development Department. Twenty two members of a user panel were mailed a covering letter, a summary of the study objectives and methods, a questionnaire exploring users’ views on the study, a pre-paid envelope to return the questionnaire; and an invitation to attend the Addenbrooke’s hospital for feedback of the results of the user opinion survey and to pose further questions to members of the study team.

The user opinion questionnaire contained 14 statements covering understanding of the study design and the acceptability of the study methods. Respondents were asked to indicate which of seven responses most closely reflected their opinion following each of the 14 statements—strongly agree, agree, neutral, disagree, strongly disagree, don’t know or do not understand.

The meeting between Addenbrooke’s patient users and the study officers (MR and PJ) was held at Addenbrooke’s Hospital on 25 June 2010 hosted by Cambridge University Hospitals NHS Foundation Trust Research and Development Department and was attended by nine members of the user panel.

The results of the user survey were returned to the National Information Governance Board Committee on 11 August 2010 permission to proceed with the study was given in a letter dated 22 September 2010.

#### Cambridge University Hospitals NHS Foundation Trust References A091700 and 09/H0306/62. Approval 2 December 2010

A Site Specific Assessment was made by the Research and Development Department and approval was given to support and sponsor the research project in accordance with the UK Department of Health Research Governance framework.

#### Medical Research Information Service of the NHS information Centre for Health and Social care. Reference MR1183. Approval 22 June 2011

Application was made in November 2010 to set up searching for death certificates of study subjects in the UK national death register, which commenced on 22 June 2011.

## Materials and Methods

### Recruitment

A list of patient admission episodes was obtained from the hospital Patient Administration System for the specialties of Care of the Elderly, General Medicine and Orthopaedics with admission date from 1 January 2005 to 31 December 2007; and admission duration ≥ 48 hours; and UK residential address.

The list of patients diagnosed with *Clostridium difficile* infection was obtained from the Hospital Infection Control Team and each case record linked to the list of total admission records from the Patient Administration System.

Admission episodes associated with *Clostridium difficile* infection were removed from the list of total admissions to produce a list of admission episodes without a record of *Clostridium difficile* infection. The lists of *Clostridium difficile* associated admissions and non-*Clostridium difficile* associated admissions were each searched for multiple admissions and the earliest episode retained and later episodes, when present, deleted. Each list was ordered by year of admission and separated into six sub-lists comprising case and non-case admissions in 2005, 2006 and 2007. Unique sequential integers were allocated to the case and non-case admission records in each of the three years, 2005, 2006 and 2007, having been sorted in alphabetical order. The admission records in each of the sorted six lists were then allocated a pseudorandom number between 0 and 1 using the 32-bit pseudorandom-number generator in Stata 11.1 [10]. Initial seeds were set for each list to ensure the process could be repeated. For each of the lists, the records with the smallest 50 pseudorandom-numbers were retained and replaced with the integers 1 to 50, 1 being given to the smallest, 2 to the next smallest, and so on.

### Sample size considerations

Sample size calculations were based on a pilot study conducted in 2006 by one of us (PJ) which showed mortality at one year of 68/287 (0.24) in non-cases; compared with 45/ 79 (0.57) in cases [11]. Planned enrolment was sufficient to demonstrate a statistically significant difference in non-case compared with case mortality at one year of 0.2 or greater, with proportion of deaths in non-cases 0.3 or less (type 1 error p = 0.05:type 2 error p = 0.20).

### Infection control arrangements and faeces testing

Infection control practice and faecal sampling protocols were constant between 2005 and 2007. Nursing staff were trained in the diagnosis of infectious diarrhoea and immediately took faecal specimens and transported them to the microbiology laboratory.

### Microbiological testing of faeces

Microbiology testing protocols were constant between 2005 and 2007. Specimens taking the shape of the faeces container were tested for *Clostridium difficile* using the cell cytotoxin assay [1].

### Case definition and microbiological test results

Faeces specimens from study cases were positive for *Clostridium difficile* by cell cytotoxin assay and negative for bacterial pathogens by culture, negative for *Cryptosporidium* and *Giardia* by microscopy, and negative for norovirus by RT- PCR.

### Collection and derivation of clinical and social variables

Clinical details for the study admission episode were abstracted by medical notes review by one of us (MR) using a structured proforma based on the International Classification of Diseases and Deaths version 10 (ICD-10); [12]. The Charlson comorbidity index without age adjustment was calculated [7]. Antibiotic and non-antibiotic medications were collected from the drug treatment charts. Interventions, surgery and recorded use of tobacco and alcohol were also collected. Microbiology test results for *Clostridium difficile* and other enteric pathogens were recorded last, in order to mask the status of the subject as being a case or non-case, as far as possible. Index of Multiple Deprivation (IMD) score for address of residence at admission was obtained from UK Government Statistics [13].

### Data Security and protection of subject identity

Data processing and protection of patient personal identifying information was undertaken in accordance with the Information Governance standards and information security standards of the National Information Governance Board, Health Protection Agency (Precursor to Public Health England), Public Health England, Cambridge University Hospitals NHS Foundation Trust and the National Health Service. Personal identifying information used in this study was kept separate from clinical and mortality data at all times, both in hard copy and in electronic media, except for data entry and data cleaning. Briefly, at completion of clinical notes review, the unique study identifier was added to all pages of the questionnaire and the cover sheet with personal identifiers was separated from the remainder of the questionnaire. Cover sheets and clinical information sections of the questionnaire were stored in separate locked filing cabinets located in separate rooms within the secure study centre. The personal identifiers of study subjects were provided to the Medical Research Information Service (MRIS) in a single encrypted file; and death certificates were returned to the study centre by MRIS in encrypted electronic files. The data base of personal identifiers and the date base of clinical information and death certificate information were held at different locations on the study centre secure local area network within separate encrypted directories. Linkage of personal identifying information and clinical and mortality data could only done by study staff in possession of the encryption keys and in computer random access memory using the unique study identifying number as key. Statistical analysis was undertaken on the anonymised data base.

### Ascertainment of deaths

The name, sex, date of birth and National Health Service number of subjects were sent to the Health & Social Care Information Centre [14] for follow up by computer searching of the national death register. Death certificates for study subjects were returned in secure electronic format and linked to individual clinical records.

### Checking case and non—case status

The presence of a positive *Clostridium difficile* microbiology result in cases and absence of such a result in non-cases was checked at clinical notes review, in the Addenbrooke’s Hospital laboratory data base and in the regional microbiology surveillance data base, to which all microbiology laboratories in the East of England routinely report. The regional laboratory surveillance data base is also completely reconciled with *Clostridium difficile* infections reported to the mandatory surveillance system for Health Care Associated infection by infection prevention and control professionals [3].

### Statistical analysis

Stata 12.1 software was used for survival analysis and version 13 for risk factor analysis [10].

#### Survival analysis

Survival was measured from date of index admission to date of certified death. Subjects for whom no death certificate was identified at the final follow up in February 2013 were treated as censored [11]. The most appropriate polynomial functions for the continuous variables were selected in single and multivariable model building.

Kaplan-Meier survival functions were compared for cases and non-cases by log-rank test, Peto-Peto-Prentice test and by Cox proportional hazards regression [11]. Variables were tested one at a time for their effect on the relationship between Group (case or non-case) and survival in Cox regression. Individual variables leading to a change of 10% or greater in the Hazard Ratio of Group; or with p-value of < 0.2 by Likelihood Ratio Test (LRT) were included in a multivariable model. A backward stepwise procedure was then undertaken removing at each step one at a time variables for which there was no substantial confounding with respect to the association between Group and survival and with LRT p value > 0.05, but retaining age and sex. Interactions between Group (case or non-case) and each of the remaining variables were investigated one at a time for significant effect modification.

The proportional hazards assumption was tested and examined graphically in both the unadjusted and adjusted single variable and final multivariable models. In those cases where proportional hazards assumption was violated, four different choices of models in the accelerated failure time metric were examined, namely, Weibull, lognormal, log logistic and generalised gamma. The choice between these was made on the basis of the one with the smallest Akaike Information Criterion (AIC).

Several model checking devices were employed. One was to calculate the Gӧnen and Heller’s K concordance coefficient to calculate the probability that the predictions and outcomes were concordant. One of the graphical checks was to check for unduly influential observations on the set of parameter estimates of the final model. The other plots all involved residuals to check on the adequacy of the model fit. The Cox-Snell residual were compared to the empirical estimate of the cumulative hazard, while the deviance residuals were plotted against time and each of the predictors in the model.

#### Risk factor analysis

Logistic regression and LRTs were used for single and multivariable case-control analysis. [15]. For continuous variables, appropriate linear, quadratic or cubic forms were identified on the logit scale. Risk factors in cases were measured from date of index admission to the date the faecal specimen tested positive for *Clostridium difficile;* and for the full duration of index admission for non-cases.

Age and sex were retained in all iterations of multivariable model building. Exposures with raised odds and p < 0.2 in single variable analysis were evaluated by introducing them in increasing order of number of missing observations and, within this, in a sequence of four blocks of variables defined by p value in single variable analysis (≤ 0.01; >0.01 to ≤0.05; >0.05 to ≥0.1; and >0.1). After a block was added, variables were removed one at a time starting with the least significant. Variables whose removal resulted in a change of 10 per cent or more of one or more of the odds ratios of other variables in the model were re-entered as significant confounders, but always retaining age and sex. The process was continued until all variables were either significant at the 10 per cent level or were substantial confounders, when the next block of variables was added. This continued until all blocks had been added, the range in the number of missing observations exhausted and no variables could be removed.

## Results

A total of 511 *Clostridium difficile* associated and 29,920 non *Clostridium difficile* associated first admission episodes were identified from which the probability samples were selected.

One hundred cases and 98 non-cases were enrolled without replacement. All subjects had residential addresses within the county of Cambridgeshire or adjacent counties in the East of England.

Time from admission to earliest positive *Clostridium difficile* specimen date in cases ranged from zero to 100 (median 14) days. In fifteen (15%) of cases this interval was less than three days, comprising day of admission, three cases; day one of admission, eight cases; and day two of admission, four cases.

All cases were treated for *Clostridium difficile* infection with oral metronidazole and/or oral vancomycin. No subjects received probiotics. *Clostridium difficile* was recorded as a cause of death or contributing to death in 17 of 99 death certificates from cases and in none of ninety six death certificates from non-cases.

### Survival analysis

#### Single variable survival analysis

Duration from index admission to death ranged from 0.02 to 8.04 years for cases; and 0.01 to 8.11 years for non-cases (Table 1).

**Table 1:** Follow-up distributions for cases and non-cases (years).

Group	Minimum	25^th^ centile	Median	75^th^ centile	Maximum
Case	0.02	0.18	0.46	3.98	8.04
Non-case	0.01	0.62	4.94	6.80	8.11

**Fig 1:**
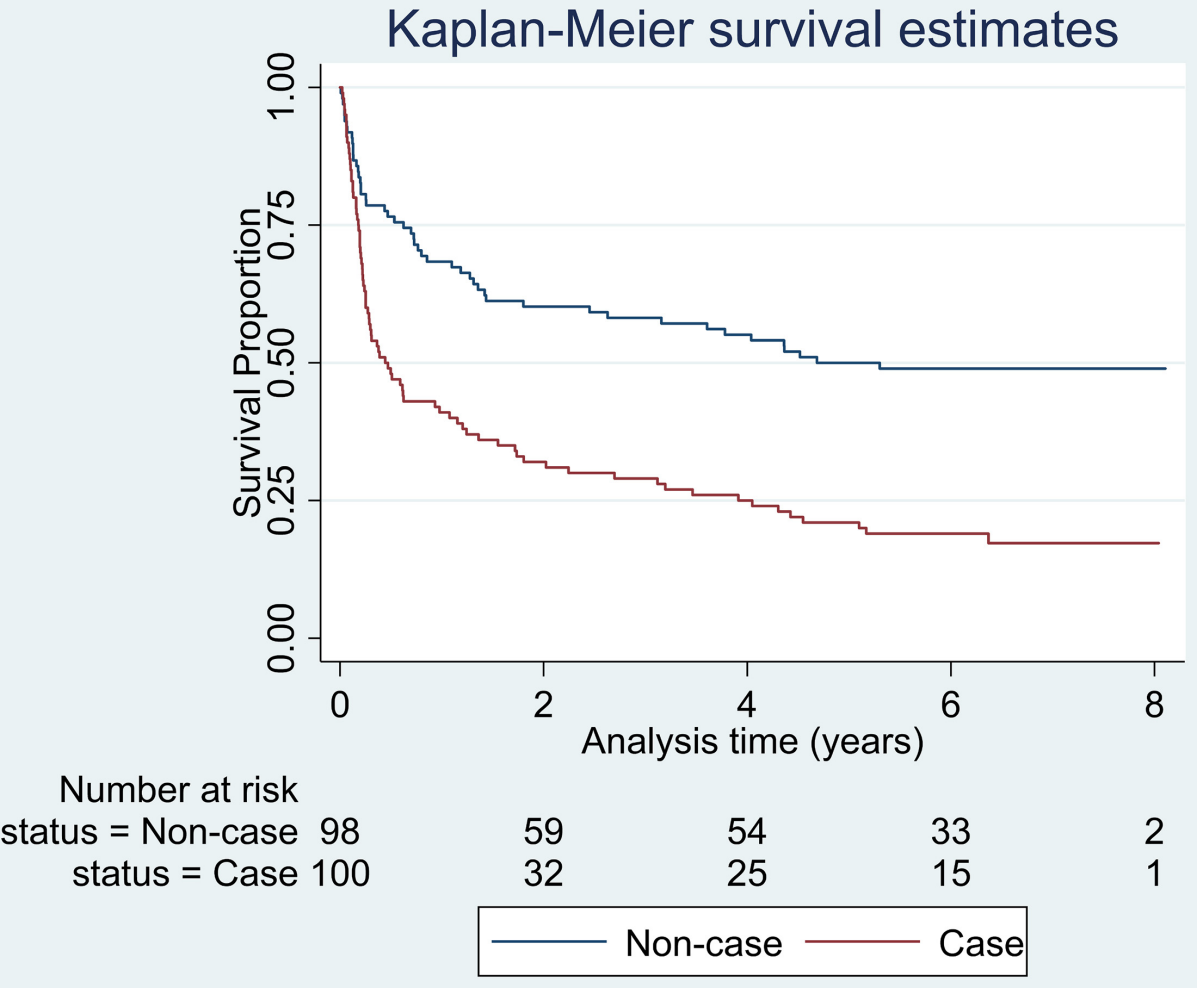
Kaplan-Meier survival estimates of non-cases and *Clostridium difficile* cases.

Survival of cases was much diminished compared to non-cases in the first year but was similar in both groups between years two and eight following index admission. The unadjusted Hazard Ratio was 2.33 (95% Confidence Interval (CI): 1.63–3.32) p <0.001 (Fig 1).

Variables identified as potential confounders of time to death and Group (case or non-case) are given in Table 2 along with Hazard Ratio for Group adjusted for each of these variables individually.

**Table 2:** Hazard Ratio for cases compared to non-cases unadjusted and adjusted for variables shown.

Variable adjusted for	Categories or five number summary	Number of individuals or value	HR	95% CI	p-value
**None (unadjusted)**	Case	100	2.33	1.63–3.32	**<0.001**
	Non-case	98			
**Age** ^a^	Number	198	1.46	1.01–2.10	0.04
	Minimum	19			
	25^th^ centile	70			
	Median	80.5			
	75^th^ centile	86			
	Maximum	98			
**Sex** ^a^	Male	87	2.30	1.61–3.29	**<0.001**
	Female	111			
**Year of admission**	2005	65	2.30	1.61–3.28	**<0.001**
	2006	67			
	2007	66			
**Month of admission**	January	24	2.34	1.59–3.44	**<0.001**
	February	22			
	March	24			
	April	18			
	May	14			
	June	11			
	July	16			
	August	18			
	September	17			
	October	11			
	November	11			
	December	12			
**Place admitted from** ^b^	Own home	160	2.20	1.53–3.17	**<0.001**
	Residential care	29			
	Another hospital	3			
	Other	4			
**Malignancy diagnosis at index admission** ^a^	Yes	31	2.39	1.67–3.40	**<0.001**
	No	167			
**Infection diagnosis other than *Clostridium difficile* at index admission** ^b^	Yes	84	2.29	1.55–3.67	**<0.001**
	No	114			
**Charlson Comorbidity index** ^a^	Number	198	2.24	1.57–3.19	**<0.001**
	Minimum	0			
	25^th^ centile	0			
	Median	2			
	75^th^ centile	3			
	Maximum	12			
**Immune compromised condition at index admission** ^b^	Yes	23	2.27	1.59–3.25	**<0.001**
	No	173			
**Haemoglobin g/dL** ^b^	Number	180	2.14	1.46–3.13	**<0.001**
	Minimum	5.6			
	25^th^ centile	10.6			
	Median	12.1			
	75^th^ centile	13.6			
	Maximum	19.4			
**Total White Cell count 10**^**9**^**/L**	Number	166	2.43	1.65–3.57	**<0.001**
	Minimum	3.1			
	25^th^ centile	7.7			
	Median	10.9			
	75^th^ centile	14.3			
	Maximum	66.3			
**Blood Glucose mmol/L**	Number	62	1.64	0.87–3.08	0.12
	Minimum	1.7			
	25^th^ centile	5.6			
	median	7.0			
	75^th^ centile	9.4			
	Maximum	70			
**Serum Creatinine umol/L**	Number	161	2.28	1.54–3.37	**<0.001**
	Minimum	5			
	25^th^ centile	72			
	Median	90			
	75^th^ centile	128			
	Maximum	512			
**Serum Urea mmol/L** ^b^	Number	145	2.27	1.50–3.44	**<0.001**
	Minimum	2.0			
	25^th^ centile	5.5			
	Median	8.0			
	75^th^ centile	14.1			
	Maximum	130.0			
**Fever≥ 38 C during Index admission** ^b^	Yes	31	2.52	1.74–3.66	**<0.001**
	No	155			
**Systolic Blood pressure ≤ 100 mmHg**	Yes	31	2.45	1.70–3.58	**<0.001**
	No	164			
**Clinical diagnosis of sepsis**	Yes	15	2.41	1.68–3.47	**<0.001**
	No	183			
**Positive blood culture**	Yes	1	2.32	1.62–3.31	**<0.001**
	No	197			
**Smoking History** ^b^	Current smoker	23	2.89	1.79–4.66	**<0.001**
	Past smoker	46			
	Never smoker	61			
**Type of alcoholic drinker**	Heavy	4	2.13	1.49–3.06	**<0.001**
	Drinker but not heavy	156			
	Non-drinker	38			
**Surgery during index admission** ^b^	Yes	51	2.50	1.75–3.57	**<0.001**
	No	145			
**Nasogastric tube inserted during index admission**^a^	Yes	28	2.30	1.58–3.33	**<0.001**
	No	167			
**Procedure other than surgery during index admission**	Yes	69	2.27	1.55–3.35	**<0.001**
	No	124			
**IMD score**	Number	198	2.37	1.66–3.39	**<0.001**
	Minimum	0.7			
	25^th^ centile	6.5			
	Median	9.6			
	75^th^ centile	14.4			
	Maximum	32.1			

^a^ indicates variables included in the final multivariable Cox Regression model

^b^ indicates variables considered in stepwise multivariable survival modelling but excluded from the final Cox Regression model

#### Multivariable survival analysis

Variables with an effect on Group (case or none-case) and survival in single variable analysis (Table 2) were admitted to a multivariable backward stepwise survival procedure. The final multivariable model consisted of the main effects of Group (case or non-case) age, sex, Charlson comorbidity index, nasogastric tube insertion during index admission and malignancy. The adjusted HR for case or non-case in the final model was 1.51 (95% CI: 1.05–2.19) p = 0.03. There were no significant interactions between Group (case or non-case) and any of the other variables in this model. Model diagnostics revealed a satisfactory final multivariable model with assumptions not being violated and robustness of findings.

### Risk factor analysis

#### Single variable risk factor analysis

*Clostridium difficile* infection was associated with age (OR 1.05 (1.03,1.07) per year p<0.001); female sex (p = 0.047); Charlson comorbidity index (p<0.001); admission with a diagnosis of infection other than *Clostridium difficile* (OR 4.63 (2.51,8.51) p<0.001); in patient treatment with fluoroquinolones (OR 2.93 (1.65,5.22) p<0.001; carbapenems OR 4.73 (1.30, 17.17) p = 0.008; intravenous vancomycin; 2.66 (1.05,6.74) p = 0.03; Proton Pump inhibitors OR 2.03 (1.13,3.64) p = 0.02; pre-admission treatment with first generation cephalosporins (OR 7.56 (0.91,62.66) p = 0.02; total number of drugs other than antibiotics prescribed during index admission OR 1.37 (1.22,1.53) per additional drug p<0.001; and having a haematological condition (OR 2.31(1.03–5.21) p = 0.04) (Table 3). Being a case also showed association with duration of admission episode to our hospital prior to the index admission (p = 0.01); and with the number of previous admissions to our hospital (OR 1.04 per additional previous admission episode p = 0.08). Significant associations were also observed for earliest:—serum urea (p<0.001); serum creatinine (p = 0.03); haemoglobin (p = 0.02); and total white cell count (p = 0.03).

**Table 3:** Single variable risk factor analysis.

Variable	Case	Non-case	OR	95% CI	p-value	Number missing observations
**Age**						
Number	100	98	1.05 per year	1.03–1.07	**<0.001**	0
Minimum	24	19				
25^th^ centile	76	58				
Median	83	75				
75^th^ centile	88	85				
Maximum	98	97				
**Sex**						
Male	37	50	0.56	0.32–0.99	**0.047**	0
Female	63	48	1.00			
**Duration of index admission to discharge or earliest positive specimen date (days)**						
Number	100	98	Quadratic function		**0.02**	0
Minimum	0	2				
25^th^ centile	7	6				
Median	14	10				
75^th^ centile	28	18				
Maximum	100	161				
**Haemoglobin g/dL**						
Number	92	88	Cubic function		**0.02**	18
Minimum	6.8	5.6				
25^th^ centile	9.9	10.9				
Median	11.6	12.9				
75^th^ centile	13.2	13.8				
Maximum	19.4	17.2				
**Total White Cell count 10**^**9**^**/L**						
Number	87	79	1.06 per unit increase	1.00–1.11	**0.03**	32
Minimum	3.1	3.2				
25^th^ centile	8.0	7.2				
Median	11.7	10.4				
75^th^ centile	14.8	13.9				
Maximum	66.3	30.5				
**Serum creatinine umol/L**						
Number	84	77	1.00 per unit increase	1.00–1.01	**0.03**	37
Minimum	5	12				
25^th^ centile	77	69				
Median	103	84				
75^th^ centile	180	111				
Maximum	512	509				
**Serum urea mmol/L**						
Number	81	64	Quadratic function		**<0.001**	53
Minimum	2.5	2.0				
25^th^ centile	6.3	4.5				
Median	9.6	6.6				
75^th^ centile	15.1	10.5				
Maximum	42.0	130.0				
**Blood glucose mmol/L**						
Number	32	30	1.00	0.97–1.04	0.8	136
Minimum	1.7	3.9				
25^th^ centile	6.0	5.4				
Median	7.0	7.1				
75^th^ centile	8.4	9.9				
Maximum	70.0	69.0				
**Charlson comorbidity index**						
Number	100	98	Cubic function		**<0.001**	0
Minimum	0	0				
25^th^ centile	1	0				
Median	2	2				
75^th^ centile	3	4				
Maximum	12	9				
**Index of Multiple Deprivation**						
< = 4.51	17	23	1.00		**0.15**	0
> 4.51–8.10	20	22	1.23	0.51–2.94		
> 8.10–10.79	16	21	1.03	0.42–2.54		
> 10.79–16.34	27	13	2.81	1.13–6.99		
> 16.34	20	19	1.42	0.59–3.46		
**Number of previous admissions**						
Number	100	98	1.04 per additional previous admission	0.99–1.08	**0.08**	0
Minimum	0	0				
25^th^ centile	1	0				
Median	3	3				
75^th^ centile	7	5				
Maximum	74	32				
**Pre-index admission duration (days)**						
No previous admission	12	27	1.00		**0.01**	1
0	24	14	3.86	1.50–9.94		
1–6	18	29	1.40	0.57–3.43		
7–13	18	13	3.12	1.16–8.35		
14–20	7	4	3.94	0.97–16.03		
21–27	8	4	4.50	1.13–17.88		
> = 28	13	6	4.88	1.49–15.90		
**Gap between pre and index admission (weeks)**						
No previous admission	12	27	1.00		**0.15**	1
< = 1 week	7	6	2.63	0.73–9.49		
>1–4 weeks	16	10	3.60	1.27–10.21		
>4 weeks– 3 months	14	10	3.15	1.09–9.08		
>3–6 months	8	10	1.80	0.57–5.70		
>6 months– 1 year	7	7	2.25	0.65–7.85		
> 1 year	36	27	3.00	1.29–6.97		
**Year**						
2005	34	31	1.00	0.46–1.81	0.9	0
2006	33	34	0.88	0.45–1.75		
2007	33	33	0.91	0.46–1.81		
**Month**						
January	12	12	1.00		0.23	0
February	13	9	1.44	0.45–4.64		
March	12	12	1.00	0.32–3.10		
April	4	14	0.29	0.07–1.12		
May	6	8	0.75	0.20–2.83		
June	6	5	1.20	0.29–5.02		
July	11	5	2.20	0.58–8.28		
August	6	12	0.50	0.14–1.77		
September	10	7	1.43	0.41–5.01		
October	5	6	0.83	0.20–3.49		
November	7	4	1.75	0.40–7.58		
December	8	4	2.00	0.47–8.46		
**Admitted from**						
Own home	80	80	1.00		**0.01**	2
Residential care	18	11	1.63	0.73–3.68		
Another hospital	0	3	0.00	n.e		
Other	0	4	0.00	n.e.		
**Smoking history at index admission**						
Smoker	37	32	1.19	0.60–2.38	0.6	68
Non-smoker	30	31	1.00			
**Alcohol drinking history at index admission**						
Drinker	25	41	0.36	0.16–0.81	**0.01**	94
Non-drinker	24	14	1.00			
**Cardiovascular condition at index admission**						
Yes	43	40	1.08	0.61–1.89	0.8	1
No	57	57	1.00			
**Hypertension at index admission**						
Yes	28	23	1.23	0.65–2.34	0.5	2
No	72	73	1.00			
**Diabetes mellitus at index admission**						
Yes	20	16	1.28	0.62–2.65	0.5	0
No	80	82	1.00			
**Respiratory condition at index admission**						
Yes	32	26	1.27	0.68–2.34	0.5	2
No	68	70	1.00			
**Gastrointestinal condition at index admission**						
Yes	33	25	1.40	0.75–2.59	0.3	2
No	67	71	1.00			
**Renal condition at index admission**						
Yes	18	12	1.54	0.70–3.39	0.3	2
No	82	84	1.00			
**Urinary tract condition at index admission**						
Yes	19	15	1.27	0.60–2.66	0.5	2
No	81	81	1.00			
**Genital condition at index admission**						
Yes	2	3	0.63	0.10–3.87	0.6	2
No	98	93	1.00			
**Central Nervous System condition at index admission**						
Yes	27	23	1.17	0.62–2.23	0.6	2
No	73	73	1.00			
**Psychiatric condition at index admission**						
Yes	25	24	1.00	0.52–1.91	>0.999	2
No	75	72	1.00			
**Metabolic condition other than diabetes mellitus at index admission**						
Yes	12	5	2.48	0.84–7.33	**0.09**	2
No	88	91	1.00			
**Endocrine condition other than diabetes mellitus at index admission**						
Yes	9	2	4.65	0.98–22.1	**0.03**	2
No	91	94	1.00			
**Trauma at index admission**						
Yes	15	16	0.88	0.41–1.90	0.7	2
No	85	80	1.00			
**Malignant condition at index admission**						
Yes	15	16	0.90	0.42–1.95	0.8	0
No	85	82	1.00			
**Skin condition at index admission**						
Yes	12	10	1.17	0.48–2.86	0.7	2
No	88	86	1.00			
**Musculoskeletal condition at index admission**						
Yes	33	32	0.99	0.54–1.79	0.96	2
No	67	64	1.00			
**Elective surgery at index admission**						
Yes	1	4	0.23	0.03–2.14	0.15	1
No	99	93	1.00			
**Haematological condition at index admission**						
Yes	21	10	2.31	1.03–5.21	**0.04**	1
No	79	87	1.00			
**Infection diagnosis other than *Clostridium difficile* at index admission**						
Yes	60	24	4.63	2.51–8.51	**<0.001**	0
No	40	74	1.00			
**Other diagnosis at index admission**						
Yes	10	9	1.06	0.41–2.74	0.9	3
No	90	86	1.00			
**Immune-compromised at index admission**						
Yes	13	10	1.35	0.56–3.23	0.5	2
No	85	88	1.00			
**Nasogastric tube inserted during index admission to discharge or earliest positive specimen date**						
Yes	13	6	2.29	0.83–6.30	0.1	0
No	87	92	1.00			
**Proton pump inhibitors during index admission to discharge or earliest positive specimen date**						
Yes	46	29	2.03	1.13–3.64	**0.02**	0
No	54	69	1.00			
**H2 antagonist during index admission to discharge or earliest positive specimen date**						
Yes	5	4	1.24	0.32–4.75	0.8	0
No	95	94	1.00			
**Percutaneous endoscopic gastrostomy during index admission to discharge or earliest positive specimen date**						
Yes	3	1	3.00	0.31–29.35	0.3	0
No	97	97	1.00			
**Surgery during index admission to discharge or earliest positive specimen date**						
Yes	28	23	1.30	0.69–2.48	0.4	2
No	70	75	1.00			
**H2 antagonist before index admission**						
Yes	1	1	0.98	0.06–15.89	0.99	0
No	99	97	1.00			
**Proton pump inhibitors before index admission**						
Yes	16	9	1.88	0.79–4.49	0.15	0
No	84	89	1.00			
**Penicillinase resistant penicillins during index admission to discharge or earliest positive specimen date**						
Yes	19	15	1.3	0.62–2.73	0.5	0
No	81	83	1.00			
**Penicillins excluding penicillinase resistant penicillins and excluding co-amoxiclav during index admission to discharge or earliest positive specimen date**						
Yes	34	27	1.35	0.74–2.48	0.3	0
No	66	71	1.00			
**Co-amoxiclav during index admission to discharge or earliest positive specimen date**						
Yes	8	3	2.75	0.71–10.7	0.12	0
No	92	95	1.00			
**Aminoglycoside during index admission to discharge or earliest positive specimen date**						
Yes	3	4	0.73	0.16–3.34	0.7	0
No	97	94	1.00			
**First generation cephalosporin during index admission to discharge or earliest positive specimen date**						
Yes	0	1	0.00	n.e.	0.23	0
No	100	97	1.00			
**Third generation cephalosporin during index admission to discharge or earliest positive specimen date**						
Yes	7	3	2.38	0.6–9.5	0.2	0
No	93	95	1.00			
**Any cephalosporin during index admission to discharge or earliest positive specimen date**						
Yes	7	4	1.77	0.50–6.25	0.4	0
No	93	94	1.00			
**Fluoroquinolone during index admission to discharge or earliest positive specimen date**						
Yes	64	37	2.93	1.65–5.22	**<0.001**	0
No	36	61	1.00			
**Macrolide during index admission to discharge or earliest positive specimen date**						
Yes	13	11	1.18	0.50–2.78	0.7	0
No	87	87	1.00			
**Carbapenem during index admission to discharge or earliest positive specimen date**						
Yes	13	3	4.73	1.30–17.17	**0.008**	0
No	87	95	1.00			
**Fusidic acid during index admission to discharge or earliest positive specimen date**						
Yes	2	0	n.e.	n.e.	0.1	0
No	98	98	1.00			
**Rifampicin during index admission to discharge or earliest positive specimen date**						
Yes	1	0	n.e.	n.e.	0.24	0
No	99	98	1.00			
**Tetracycline during index admission to discharge or earliest positive specimen date**						
Yes	2	0	n.e.	n.e.	0.1	0
No	98	98	1.00			
**Trimethoprim during index admission to discharge or earliest positive specimen date**						
Yes	3	3	0.98	0.19–4.97	0.98	0
No	97	95	1.00			
**Glycopeptide intravenous (vancomycin) during index admission to discharge or earliest positive specimen date**						
Yes	17	7	2.66	1.05–6.74	**0.03**	0
No	83	91	1.00			
**Any antibiotic except oral vancomycin and oral metronidazole during index admission to discharge or earliest positive specimen date**						
Yes	83	60	3.09	1.6–5.99	**<0.001**	0
No	17	38	1.00			
**Number of antibiotics except oral vancomycin and oral metronidazole during index admission to discharge or earliest positive specimen date**						
Number	100	98	1.66 per antibiotic	1.28–2.15	**<0.001**	0
Minimum	0	0				
25^th^ centile	1	0				
Median	2	1				
75^th^ centile	2	2				
Maximum	6	5				
**Number of drugs given other than antibiotics during index admission to discharge or earliest positive specimen date**						
Number	100	98	1.37 per drug	1.22–1.53	**<0.001**	0
Minimum	0	0				
25^th^ centile	6	3				
Median	8	5				
75^th^ centile	10	7				
Maximum	20	14				
**Penicillinase resistant penicillins before index admission**						
Yes	1	1	1.01	0.06–16.40	0.99	7
No	94	95	1.00			
**Penicillins excluding penicillinase resistant penicillins and co-amoxiclav before index admission**						
Yes	5	5	1.01	0.28–3.61	0.99	7
No	90	91	1.00			
**Co-amoxiclav before index admission**						
Yes	0	3	0.00	n.e.	**0.04**	7
No	95	93				
**Fluoroquinolone before index admission**						
Yes	6	8	0.74	0.25–2.23	0.6	7
No	89	88	1.00			
**Macrolide before index admission**						
Yes	2	4	0.49	0.09–2.77	0.4	7
No	93	92	1.00			
**First generation cephalosporin before index admission**						
Yes	7	1	7.56	0.91–62.66	**0.02**	7
No	88	95	1.00			
**Second generation cephalosporin before index admission**						
Yes	1	0	n.e.	n.e.	0.24	7
No	94	96	1.00			
**Any cephalosporin before index admission**						
Yes	8	1	8.74	1.07–71.27	**0.01**	7
No	87	95	1.00			
**Trimethoprim before index admission**						
Yes	2	1	2.04	0.18–22.92	0.6	7
No	93	95	1.00			
**Nitrofurantoin before index admission**						
Yes	1	0	n.e.	n.e.	0.23	7
No	94	96	1.00			
**Any antibiotic given before index admission**						
Yes	24	20	1.23	0.63–2.41	0.5	0
No	76	78	1.00			
**Number of drugs given other than antibiotics before index admission**						
Number	100	98	1.14 per drug	1.02–1.26	**0.01**	0
Minimum	0	0				
25^th^ centile	2	2				
Median	4	3				
75^th^ centile	6	5				
Maximum	10	10				

#### Multivariable risk factor analysis

*Clostridium difficile* infection was independently associated with age OR 1.05 (1.02, 1.09) per year p<0.001; diagnosis of infection on admission OR 5.79 (2.19, 15.25) p<0.001; number of non-antibiotic medications during admission (1.28 per drug (1.10–1.47) p< 0.001); Index of Multiple Deprivation (p = 0.05); and prior to index admission, with first generation cephalosporins OR 11.59 (0.66, 202.16) p = 0.06; and number of drugs other than antibiotics OR 1.14 (1.02,1.26) p = 0.01 (Table 4). Metabolic diagnosis other than diabetes; and haematological condition were also present in the final model, but with confidence intervals that included unity. Number of previous admission to our hospital and duration of pre-index admission also met our criteria for inclusion in the final multivariable model, but did not reach the standard 5% level of statistical significance.

**Table 4:** Final multivariable logistic regression risk factor analysis n = 186.

Variable	OR	95% CI	p-value
**Age**	1.05 per year	1.02–1.09	**<0.001**
**Sex**			
Male	0.56	0.24–1.30	0.17
Female	1.00		
**Number of drugs given other than antibiotics during index admission to discharge or earliest positive specimen date**	1.28 per drug	1.10–1.47	**<0.001**
**Diagnosis of infection other than *Clostridium difficile* at index admission**			
Yes	5.79	2.19–15.25	**<0.001**
No	1.00		
**Number of previous admissions**	1.03 per additional previous admission	0.98–1.08	0.23
**Index of Multiple Deprivation**			
< = 4.51	1.00		**0.05**
> 4.51–8.10	1.66	0.44–6.29	
> 8.10–10.79	1.31	0.35–4.90	
> 10.79–16.34	5.82	1.39–24.28	
> 16.34	4.41	1.06–18.40	
**Fluoroquinolone during index admission to discharge or earliest positive specimen date**			
Yes	1.67	0.70–3.98	0.25
No	1.00		
**Pre-index admission duration (days)**			
No previous admission	1.00		0.19
0	5.26	1.25–22.15	
1–6	1.72	0.43–6.84	
7–13	1.38	0.31–6.23	
14–20	3.51	0.42–29.32	
21–27	4.54	0.57–36.33	
> = 28	4.39	0.70–27.69	
**Haematological condition at index admission**			
Yes	2.59	0.70–9.52	0.14
No	1.00		
**Admitted from**			
Own home	1.00		0.06
Residential care	0.81	0.26–2.52	
Another hospital	0.00	n.e.*	
Other	0.00	n.e.	
**Metabolic condition other than diabetes mellitus at index admission**			
Yes	8.06	0.97–66.95	**0.04**
No	1.00		
**First generation cephalosporin before index admission**			
Yes	11.59	0.66–202.16	0.06
No	1.00		

*n.e. = not estimable

## Discussion and Conclusions

We estimated a 51 per cent increase in all-cause mortality attributable to *Clostridium difficile* infection (Hazard Ratio 1.51 (95% CI: 1.05–2.19) p = 0.03) after adjustment for age, sex, Charlson comorbidity index, nasogastric tube insertion during index admission and diagnosis of malignancy in an historic cohort of 100 cases and 98 non-cases followed for up to eight years from index admission to date of certified death. The unadjusted estimate of attributable mortality was over two fold higher (Hazard Ratio 2.33 (1.63–3.32) p<0.001). The excess risk of death was confined to the first year following index admission.

The variables which met our selection criteria for admission to the stepwise survival modelling procedure were urea and haemoglobin concentrations, malignant diagnosis, the Charlson comorbidity index, tobacco use, surgery, fever, nasogastric tube insertion, being immune compromised, having an infection diagnosis at admission and setting from where admitted. Of these, only the Charlson comorbidity index, having a malignant condition and insertion of nasogastric tube were identified as significant confounders of the relationship between survival and being a case or non-case along with age and sex and were included in the final multivariable survival model [16].

The Charlson Comorbidity Index satisfactorily expressed the comorbidities that we measured for their effect on survival with the exception of diagnosis of malignancy and nasogastric tube insertion. Nasogastric tube insertion may have acted directly on survival or be a further marker of multiple comorbidity. Insertion of a nasogastric tube is also a recognised risk factor for acquisition of *Clostridium difficile* infection and was associated with raised odds in our single variable risk factor analysis although with a confidence interval including unity ((OR 2.29(0.83,6.30) p = 0.1). These risks should be considered before nasogastric intubation.

Our findings are noteworthy because our *Clostridium difficile* cases were representative of the case mix of incident cases at our hospital with only 13 (13%) of cases having a clinical diagnosis of sepsis and none diagnosed with toxic megacolon or having undergone colectomy, which are features of severe *Clostridium difficile* Associated Disease (CDAD). Survival is likely more compromised in severe *Clostridium difficile* infections associated with major outbreak strains [6;17–20].

Our subjects differed by whether they had been infected with *Clostridium difficile* or not. Despite comprehensive searching of our microbiology surveillance system, no instance of a non-case acquiring *Clostridium difficile* infection before or after index admission, or a case having a positive specimen prior to or after index admission was identified. Cases and non-cases could therefore be regarded as fixed cohorts and were representative of the population of hospitalized patients from which they were drawn [11;16]. We measured, evaluated and adjusted for demographic, clinical and social variables, so that excess all-cause mortality in cases compared to non-cases could be attributed to *Clostridium difficile* infection [16]. This approach to estimating attributable mortality is independent of the certified cause of death. Death certification depends on the judgement of certifying physicians on the contribution of individual medical conditions in the complex pathway of events leading to an individual death and is subject to complex biases [21]. The limitation of cause specific death registration for *Clostridium difficile* is suggested in our study by *Clostridium difficile* having been recorded in only about a fifth of the death certificates of our study cases.

Sepsis is a recognised complication of *Clostridium difficile* associated Disease (CDAD). Sepsis has been defined as “a systemic inflammatory response to infection, which is a progressive and injurious. process, which includes sepsis associated organ dysfunction” [22]. Our data set included variables which are components of this sepsis definition including clinical diagnosis of sepsis, white blood cell count, fever, serum creatinine, serum urea, low systolic blood pressure and positive blood culture. Although each of these variables resulted in an effect on the Hazard Ratio in single variable survival analysis none met our criteria for inclusion in our final multivariable survival model.

We recorded smoking and alcohol use at notes review, but these records were incomplete in a high proportion of subjects. We also obtained the Index of Multiple Deprivation of home address at index admission. Although these measures were partial and likely to be incompletely precise, each of these variables did modify the relationship between survival and being a case or non-case in single variable analysis with adjusted Hazard Ratios of 2.89 (1.79, 4.66) for smoking; 2.13 (1.49, 3.06) for alcohol; and 2.37 (1.66, 3.39) for Index of Multiple Deprivation (Table 2). However, alcohol, tobacco and Index of Multiple Deprivation did not meet our selection criteria for inclusion in our final multivariable survival model. We therefore believe that it is unlikely that the decreased survival which we attribute to *Clostridium difficile* infection could be overestimated as a consequence of residual confounding by alcohol, smoking or social deprivation.

We also undertook a risk factor analysis of our study cohort to determine to what degree this resembled case-control studies reported from other health systems. Risk factors for *Clostridium difficile* in our study cohort were unremarkable comprising antibiotic exposure, which was greater the broader the spectrum of antibiotics, gastric acid suppressants, haematological conditions and uraemia, which have been widely reported before [1;23–25]. Our estimate of *Clostridium difficile* attributable mortality is also consistent with short term (one year) hospital based follow up studies reported from another English Hospital [26], and a teaching hospital in Austria in which patients hospitalised for enteric infections other than *Clostridium difficile* were used as a reference population [27]. These observations suggest our measure of *Clostridium difficile* attributable mortality in a routine case mix of hospitalised patients may be more widely generalizable.

Being admitted with an active infection other than *Clostridium difficile* was associated with high independent odds of being a *Clostridium difficile* case (OR 5.79(2.19,15.29)). This variable was defined as having a diagnosis of infection at, or being prescribed antibiotics within eight weeks of index admission. Although these high odds are not surprising, they show that patients at greatest risk of *Clostridium difficile* infection are easily recognised at admission and may be prioritised for preventive efforts, which could include simple operational considerations such as seeking to minimise their bed and ward movements during admission as far as possible.

Fifteen (15%) of our cases had a positive specimen for *Clostridium difficile* less than four days following admission and on this basis it has been suggested that attribution of acquisition of *Clostridium difficile* infection may be made to an exposure occurring elsewhere from the hospital to which the patient has been admitted [3]. Most of our subjects had had multiple (median 3) previous admissions to our hospital. *Clostridium difficile* infection showed evidence of association with duration of pre-index admission (p = 0.01) and with total number of previous admissions to our hospital (OR 1.04 (0.99,1.08) p = 0.08) per additional previous admission in single variable analysis (Table 3). Both variables were retained in our final multivariable risk factor model although they were no longer significant (Table 4). These observations may suggest that a short lead time between admission date and date of a positive specimen for *Clostridium difficile* may not exclude the possibility of infection having been acquired at an earlier admission to the same hospital.

The odds of *Clostridium difficile* infection were increased with the total number of medications additional to antibiotics prescribed. This may be explained by correlation with comorbidity, or possibly by a direct effect of multiple medications disturbing innate and local protective mechanisms in the upper gastro intestinal tract, such as by inducing irritation of mucosal surfaces. This risk could be minimised by parsimonious prescribing and favours development and use of minimally irritant presentations of drugs.

To the authors’ knowledge this is the first follow up study comparing survival of a probability sample of *Clostridium difficile* cases diagnosed by the Gold Standard cell cytotoxin assay, and a probability sample of non- infected hospitalised patients as a reference population, linking clinical details from hospital records to death certificates obtained by active computer searching of a national death register.

We have shown a fifty per cent excess mortality attributable to *Clostridium difficile* occurring during the first year following infection in a case mix of hospitalised patients likely to be representative of endemic *Clostridium difficile* infections in the English National Health Service (NHS).

Our results highlight the continued importance for the NHS and other health systems of sustaining and improving existing methods of control of *Clostridium difficile* infection by steps to reduce direct and indirect faeco oral cycling of infection between patients. This requires safe disposal of faeces by good provision of lavatories, sluices, wash basins and safe handling and transport of contaminated linens to laundry, as well as adequate capacity and practice for prompt isolation of cases of infectious diarrhoea and application of enteric nursing precautions. Sustained effort is required in design and materials sciences to make hospital environments and artefacts easier to decontaminate and clean [28] as well as maintaining sound antibiotic stewardship.

Our findings strongly support continued priority being given to research for new preventive measures and treatments for *Clostridium difficile* including emerging therapies such as faecal transplantation and kindred interventions to restore a more normal gut flora [29–31].
